# Long‐term sound and movement recording tags to study natural behavior and reaction to ship noise of seals

**DOI:** 10.1002/ece3.4923

**Published:** 2019-02-06

**Authors:** Lonnie Mikkelsen, Mark Johnson, Danuta Maria Wisniewska, Abbo van Neer, Ursula Siebert, Peter Teglberg Madsen, Jonas Teilmann

**Affiliations:** ^1^ Department of Bioscience Aarhus University Roskilde Denmark; ^2^ Sea Mammal Research Unit University of St. Andrews St. Andrews UK; ^3^ Department of Bioscience Aarhus University Aarhus C Denmark; ^4^ Hopkins Marine Station Stanford University Stanford California; ^5^ Institute for Terrestrial and Aquatic Wildlife Research (ITAW) University of Veterinary Medicine Hannover Foundation Germany; ^6^ Aarhus Institute for Advanced Studies Aarhus University Aarhus C Denmark

**Keywords:** anthropogenic noise, behavioral response, biologging, DTAG, exposure rates, gray seal, harbor seal, long‐duration acoustic dataloggers

## Abstract

1. The impact of anthropogenic noise on marine fauna is of increasing conservation concern with vessel noise being one of the major contributors. Animals that rely on shallow coastal habitats may be especially vulnerable to this form of pollution.
2. Very limited information is available on how much noise from ship traffic individual animals experience, and how they may react to it due to a lack of suitable methods. To address this, we developed long‐duration audio and 3D‐movement tags (DTAGs) and deployed them on three harbor seals and two gray seals in the North Sea during 2015–2016.
3. These tags recorded sound, accelerometry, magnetometry, and pressure continuously for up to 21 days. GPS positions were also sampled for one seal continuously throughout the recording period. A separate tag, combining a camera and an accelerometer logger, was deployed on two harbor seals to visualize specific behaviors that helped interpret accelerometer signals in the DTAG data.
4. Combining data from depth, accelerometer, and audio sensors, we found that animals spent 6.6%–42.3% of the time hauled out (either on land or partly submerged), and 5.3%–12.4% of their at‐sea time resting at the sea bottom, while the remaining time was used for traveling, resting at surface, and foraging. Animals were exposed to audible vessel noise 2.2%–20.5% of their time when in water, and we demonstrate that interruption of functional behaviors (e.g., resting) in some cases coincides with high‐level vessel noise. Two‐thirds of the ship noise events were traceable by the AIS vessel tracking system, while one‐third comprised vessels without AIS.
5. This preliminary study demonstrates how concomitant long‐term continuous broadband on‐animal sound and movement recordings may be an important tool in future quantification of disturbance effects of anthropogenic activities at sea and assessment of long‐term population impacts on pinnipeds.

## 1 INTRODUCTION

Growing industrialization of the marine environment is resulting in habitat changes and increasing marine defaunation (McCauley et al., 2015; Richardson, Greene, Malme, & Thomson, 1995). A greater awareness of increasing levels of anthropogenic noise has prompted studies to understand and mitigate their potential negative impacts on marine life (Hildebrand, 2005). While many recent studies have sought to address the effects of underwater noise on cetaceans (Nowacek, Thorne, Johnston, & Tyack, 2007), comparatively less is known about exposure and reactions to noise in pinnipeds while at sea. Like cetaceans, pinnipeds have sensitive underwater hearing; their full hearing range extends from a few hundred Hz to 70–80 kHz (Cunningham & Reichmuth, 2016; Hemilä, Nummela, Berta, & Reuter, 2006). They rely on sound for communication (Mathevon, Casey, Reichmuth, & Charrier, 2017; Van Parijs, Hastie, & Thompson, 1999), predator detection (Deecke, Slater, & Ford, 2002), and possibly also for navigation and listening for prey (Schusterman, Levenson, Reichmuth, & Southall, 2000). Pinnipeds have been found to respond strongly to underwater tone pulses at 8–45 kHz in captivity (Götz & Janik, 2010; Kastelein et al., 2015; Kastelein, Heul, Terhune, Verboom, & Triesscheijn, 2006a; Kastelein, Heul, Verboom, Triesscheijn, & Jennings, 2006b) and to sounds from seismic surveys (Harris, Miller, & Richardson, 2001) and pile driving (Russell et al., 2016) in the wild.

A major technical challenge in assessing the impact of noise on marine fauna is that of sampling the noise levels routinely experienced by animals in the wild and simultaneously the animals’ natural behavior. While controlled experiments have led to substantial progress in evaluating responses of free‐ranging marine mammals to impulsive noise sources such as sonar or air guns (Miller et al., 2009; Tyack et al., 2011; van Beest et al., 2018), few studies have examined the effects of continuous noise, including ship noise, which may dominate the ambient noise level in coastal areas and near shipping lanes (Wisniewska et al., 2018). Ship noise may be especially relevant to coastal seals that rely on periodic land‐based resting (hauling out) and therefore spend much of their lives in coastal habitats that strongly overlap with marine traffic.

**Figure 1:**
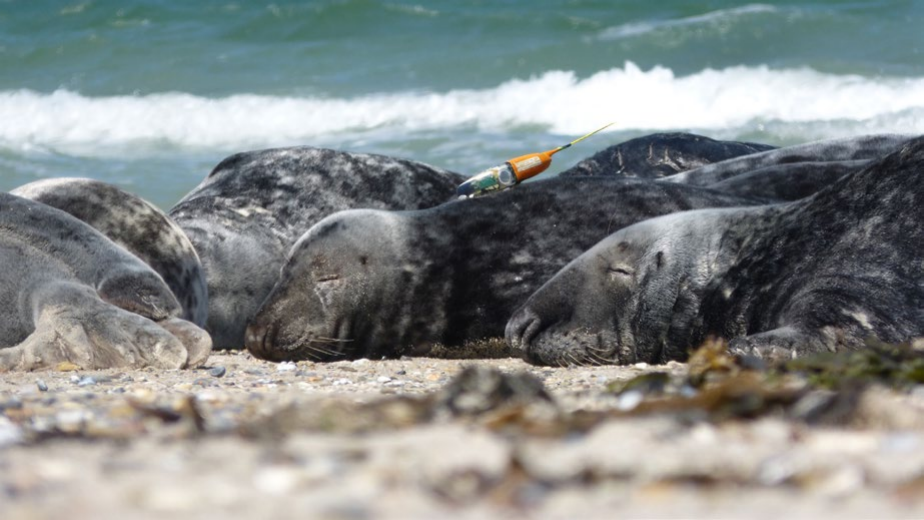
Sleeping gray seal with a DTAG3 on Helgoland May 2015. Photo: Sabine Schwarz

Pinniped at‐sea behavior has been studied extensively using a variety of biologging technologies. Tracking devices based on Argos, GPS, or VHF have provided insight into horizontal movement patterns, whereas time–depth recorders, alone or integrated in positioning devices, have been used to study dive pattern (Carter, Bennett, Embling, Hosegood, & Russell, 2016). In coastal species, such as gray seals (*Halichoerus grypus, *Figure 1) and harbor seals (*Phoca vitulina)*, at‐sea behavior has mainly been described based on 2D dive profiles, where dive behaviors are classified as traveling dives (V‐shaped dives), foraging (U‐shaped dives), or resting dives (skewed dives), as well as resting at the surface (Russell et al., 2015; Thompson, Hammond, Niceolas, & Fedak, 1991), which in some cases has been validated through camera use (Heaslip, Bowen, & Iverson, 2014).

Low‐sampling‐rate position and dive data can generally be transmitted by radio, through mobile phone networks or satellite, enabling long‐term data collection with tags that do not need to be retrieved (McConnell et al., 2004). However, the rate at which data are collected needs to match the scale of movement of a particular species and the behavior of interest. Using dive shapes to distinguish search, resting and foraging relies on assumptions about behavior which may be too simple (Ramasco, Biuw, & Nilssen, 2014). While position data have been used to infer traveling and foraging using area‐restricted search (ARS) or first passage time (FPT) analyses, these inferences have received limited validation. New state‐space models (SSM), like hidden Markov models (HMM), have the potential of integrating 3D movements and environmental parameters, increasing the power to distinguish behaviors. However, the accuracy of such models to quantify various states of behavior is highly influenced by data resolution (Carter et al., 2016). Higher‐sampling‐rate sensors, and in particular accelerometers, have proven to be useful for interpreting fine‐scale dive behaviors such as prey capture events (Gallon et al., 2013; Heerah, Hindell, Guinet, & Charrassin, 2014; Volpov et al., 2015). The challenge in high‐resolution data (from sound, cameras, or accelerometers) is the large amount of data that cannot be transmitted by radio, requiring instead that data are stored on board the tag which must be physically recovered. It is, therefore, challenging to obtain high‐resolution data over long periods of time and in remote areas. Moreover, even when tags can be recovered, new methods are needed for the efficient analysis of the complex multi‐sensor datasets returned by these devices.

To assess the effects of anthropogenic noise sources, the received noise levels and detailed animal behavior must be estimated simultaneously (Nowacek et al., 2007). Studies of pinniped responses to noise have largely relied on visual or video surveillance of animals at the surface or when hauled out (Andersen, Teilmann, Dietz, Schmidt, & Miller, 2012; Blackwell, Lawson, & Williams, 2004; Harris et al., 2001) combined with noise propagation modeling with no direct measurement of noise exposure. A more direct approach is to sample the acoustic environment of the animal using a sound recording tag (Johnson, Aguilar Soto, & Madsen, 2009). Despite being first developed to study the effects of sound exposures on the dive behavior of deep‐diving elephant seals (Burgess, Tyack, Boeuf, & Costa, 1998; Costa et al., 2003; Fletcher, Boeuf, Costa, Tyack, & Blackwell, 1996), sound and movement recording tags have not been widely used on smaller pinnipeds, due perhaps to the large size of earlier versions of these tags. More recently, compact sound recording tags, such as the DTAG (Johnson & Tyack, 2003), that combine sound recordings with high‐bandwidth movement sensors have been widely used to study noise impacts on cetaceans. These tags, which are typically attached with suction cups, have been used to link short‐term behavioral responses to specific noise sources such as military sonar (DeRuiter et al., 2013), air guns used in oil prospection (Madsen et al., 2006; Miller et al., 2009), and ship noise (Aguilar Soto et al., 2006; Nowacek, Johnson, & Tyack, 2004; Wisniewska et al., 2018). DTAG deployments on cetaceans have so far been limited by the duration of suction cup attachments, as well as memory and/or battery capacity constraints related to the small tag size, with a typical maximum recording time of less than two days at high sampling rates (Johnson et al., 2009). Such short durations are not ideal for assessing exposures and responses to opportunistic noise sources such as vessel passes. However, advances in low‐power electronic technology now allow for increased battery and memory capacity, resulting in extended periods of continuous recording of three or more weeks, while simultaneously reducing tag size. Seals are ideal candidates for these longer‐term devices, as the tags can be glued to the fur of the animal.

Here, we present initial results from newly developed, long‐term high‐resolution sound and movement DTAGs deployed on harbor and gray seals, representing the first multi‐week, continuous broadband sound recordings from any marine animal. We demonstrate the potential of such data for quantifying individual noise exposure in synchrony with the fine‐scale behaviors of the animal, enabling the identification of noise‐induced behavioral alterations together with their consequences for individual time–energy budgets. For visual verification, we furthermore combined simultaneous video and accelerometry recordings on wild seals.

## 2 MATERIALS AND METHODS

### 2.1 DTAG deployment

During 2015–2016, three harbor seals and two gray seals were captured for tag attachment at Lorenzensplate (N 54.4393, E 8.6419) in the German Wadden Sea and at Helgoland (N 54.1886, E 7.9117) in Germany, respectively (Table 1). The study was approved under permit number Az V312‐ 72241.121‐19 (70‐6/07) and V244‐3986/2017 (17‐3/14) of the Ministry of Energy, Agriculture, Environment and Rural Areas of Schleswig‐Holstein, Germany.

**Table 1:** Tag deployment summary. Age class was a subjective judgement based on size of the animal

Animal ID	hs15_069a	gs15_139a	gs15_139b	hs15_278a	hs16_265c	2016–138070	2017–6421
Species	Harbor seal	Gray seal	Gray seal	Harbor seal	Harbor seal	Harbor seal	Harbor seal
Tag version	DTAG3	DTAG3	DTAG3	DTAG3	DTAG4	Camera tag	Camera tag
Sex	Male	Female	Male	Female	Female	Male	Female
Weight (kg)	85	100	144	66	62	71	58
Std. length (cm)	149	176	144	126	148	138	121
Age class	Adult	Juvenile	Subadult	Adult	Adult	Adult	Adult
Deployment location	Lorenzensplate	Helgoland	Helgoland	Lorenzensplate	Lorenzensplate	Bosserne	Bosserne
Tagging date	10‐03‐2015	19‐05‐2015	19‐05‐2015	5‐10‐2015	21‐09‐2016	12‐08‐2016	27‐07‐2017
Recording period on the seal	10‐03‐2015‐ 24‐03‐2015	19‐05‐2015‐ 02‐06‐2015	19‐05‐2015‐ 04‐06‐2015	5‐10‐2015‐ 19‐10‐2015	21‐09‐2016‐ 11‐10‐2016	13‐08‐2016 06:00‐08:30	28‐07‐2017 08:20–10:50
Duration of usable data	336 hr (14 days)	336 hr (14 days)	377 hr (15.7 days)	336 hr (14 days)	521 hr (21.7 days)	2.5 hr of video	2.5 hr of video
Proportion of time spent hauled out (on land/partly submerged)	42.3% (28.7/13.6%)	24% (19.4/4.6%)	36% (29.2/6.8%)	6.6% (6.4/0.1%)	Data not available yet	–	–
Proportion of at‐sea time spent resting (mean submersion time/*SD*)	8.1% (5.3/1.4 min)	7.2% (6.2/2.2 min)	5.3% (5.2/1.4 min)	12.4% (4.0/1.2 min)	Data not available yet	–	–
Proportion of at‐sea time exposed to vessel noise	10.6%	2.2%	20.5%	6.6%	Data not available yet	–	–
Vessel noise events (min/max duration)	30 (1.8/330 min)	17 (1/81 min)	74 (1.9/170 min)	34 (1.2–173 min)	41		

Harbor seals were caught when hauled out on sand banks by surrounding the seals using a large net (3 m × 200 m) deployed from two boats and then dragging the net manually onshore, where the seals were transferred into tube nets and manually restrained for handling and tagging (Jeffries, Brown, & Harvey, 1993). Gray seals were caught using a lightweight pole‐net made of carbon fiber wind surfer masts and nylon net (mesh size: 2 × 2 cm). Seals were caught at low tide when they were too far up the beach to reach the water before being enclosed in the pole‐net (Arcalís‐Planas et al., 2015). Animals were restrained in the nets for approx. 30 min to determine sex, weight, and length and to attach the tags. A blood sample was also taken for health assessment. Two versions of the tag were used. The 2015 tag (DTAG‐3) contained a syntactic foam float and had an integrated Argos transmitter and VHF beacon (Figure 1). A more compact version of the tag (DTAG‐4) was used in 2016, and this was attached to a high‐pressure closed cell foam float containing an Argos transmitter with an integrated low‐power UHF beacon (SPOT 6, Wildlife Computers, Seattle, USA). The complete DTAG‐3 package measured 55 × 37 × 205 mm and weighed 325 g in air, while the DTAG‐4 measured 40 × 33 × 180 mm and weighed 206 g. Both tags were approx. 20 g buoyant in water. The tags were mounted on an aluminum release plate (1‐mm thick), which in turn was glued to the fur on the seal's upper back with two‐component epoxy (Ergo 7211). For the DTAG‐3, a 2.5 mm magnesium nut, a stainless steel washer, and a 5 mm nylon pin (glued to the float) held the tag to the aluminum plate. The tag was released from the plate when the nut corroded after approx. 3–4 weeks. In DTAG‐4, nickel–chromium wires on each side of the tag secured the tag to the aluminum plate. After a preprogrammed time, the wires were made anodic by a current from the battery to cause their rapid corrosion. One of the four DTAG‐3 and the DTAG‐4 deployment did not release as planned and remained attached to the animals for several weeks. These tags were brought to the coast by water currents where they were found by local residents. The remaining tags were recovered by boat using Argos to get an approximate position (i.e., within a radius of a few kilometers) followed by VHF tracking.

Both versions of the DTAG contained sensors for sound, pressure (depth), acceleration, magnetic field, and GPS. Sound was sampled from a single 10‐mm‐diameter end‐capped cylindrical piezo‐ceramic hydrophone at a rate of 240 kHz (DTAG‐3) or 192 kHz (DTAG‐4) with 16‐bit resolution. Sound data were decimated within the tag by a factor of 2 (DTAG‐3) or 3 (DTAG‐4) followed by loss‐less compression (Johnson, Partan, & Hurst, 2013) before being stored in memory, resulting in a stored sampling rate of 120 kHz (DTAG‐3) or 64 kHz (DTAG‐4). To reduce the chance of clipping due to flow noise when the seal is swimming, a one‐pole high‐pass filter was included with a cut‐off frequency of 100 Hz, resulting in a recording bandwidth (−3‐dB bandwidth) from 100 Hz to 51 kHz and 100 Hz to 27 kHz for the DTAG‐3 and DTAG‐4, respectively. Sensors comprising a triaxial accelerometer, a triaxial magnetometer, and a pressure transducer were sampled with 16‐bit resolution at 200 Hz per channel in DTAG‐3, and at 200 Hz (acceleration) and 50 Hz (magnetometer and pressure) in DTAG‐4. The tags also included a snapshot GPS which made 64 ms acquisitions of available GPS satellite signals when the animal surfaced. This sensor functioned poorly due to electrical interference on the DTAG‐3 but was operational on the DTAG‐4. GPS processing was performed after recovery of the tag using custom software and Internet‐published satellite almanacs. The sound, sensor, and GPS data were stored on a 64‐GB flash memory array in the tags.

### 2.2 Camera tag deployment

The camera tag comprised a triaxial accelerometer and dive logger (200 Hz sampling rate, “OpenTag,” Loggerhead Instruments, Sarasota, FL, USA), a small digital camera (30 fps, DVL 200, Little Leonardo, Tokyo, Japan), an Argos satellite transmitter (SPOT 5), and a VHF transmitter, all enclosed in a high‐pressure closed cell foam. The accelerometer/dive logger and the video were synchronized prior to deployment, and timing synchrony was subsequently verified by comparing the timing of surface intervals in the dive and video data. This tag (approx. 55 × 30 × 150 mm, weight in air 230 g) was deployed on an adult male harbor seal at “Bosserne” (N 55.9367; E 10.7757), Kattegat, Denmark. A second deployment on a female harbor seal was conducted at the same location but with the tag package reduced in size and the Argos and VHF transmitters replaced with a SPOT 6 Argos transmitter (tag dimensions approx. 35 × 35 × 160 mm, weight in air 170 g). Both tags were slightly buoyant in water to enable recovery. On both occasions, the camera was set with a time delay to start recording on the morning following tag attachment in an effort to avoid sampling disturbed behavior related to the handling of the animal. The camera tag was mounted to an aluminum plate precoated with standard construction adhesive (SMP‐38) which was in turn attached to the back of the seal. The coating was necessary to facilitate the use of super glue (Loctite 422) between the seal and the plate. The tag was held onto the aluminum plate by a 1 mm magnesium nut, a stainless steel washer, and a 5 mm nylon pin that was glued to the float. The nut corroded after 2 days permitting the tag to release from the animal.

### 2.3 Data analysis

Data analyses were performed in Matlab R2013b (MathWorks Inc.). Sound exposure was quantified as one‐third octave band levels (TOLs), that is the root mean square (RMS) sound pressure level in one‐third octave bands roughly approximating the filter bank of the mammalian auditory system (Richardson et al., 1995). To reduce the contribution of noises made by the tagged animal (e.g., due to movement), the TOLs were computed in three steps similar to the method in Wisniewska et al. (2018). First, successive 4,096 (DTAG‐3) or 2048 (DTAG‐4) point FFTs (Hann window, 50% overlap) were computed of the sound recording giving a frequency resolution of 29 Hz and 31 Hz, respectively. The power in the FFT bins falling between 3 and 20 kHz was summed to give a broadband noise estimate that is largely free of low‐frequency flow noise with a sampling rate of 58 or 62 Hz (i.e., one estimate per 50% overlapped FFT). The lower 10th percentile of this broadband noise estimate over 30 s intervals was computed, and FFTs that had a noise estimate below the 10th percentile were identified. The spectral power of these FFTs was averaged to give a 30 s ambient noise spectrum that is robust to sound transients. Finally, TOLs were estimated from the 30 s average spectra by combining the power in FFT bins which fell within each third‐octave band and converting to a received level in dB re 1µPa (RMS) by correcting for the sensitivity of the tags of −176 dB re V/µPa.

**Figure 2:**
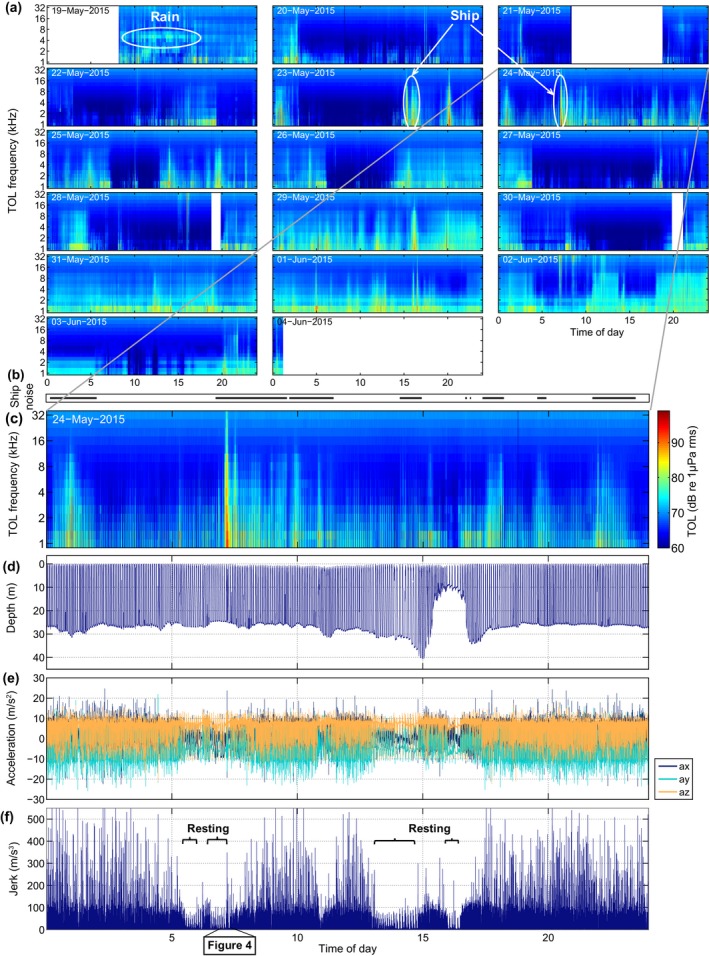
(a) Audio from the full DTAG‐3 deployment on gray seal gs15_139b, displayed as RMS sound pressure level in third‐octave bands (TOL, 1–32 kHz) averaged over 30 s. The tag did not collect data during the three uniform white intervals due to technical issues. (b–f) Data from a single day, where (b) indicates when ship noise is audible; (c) third‐octave levels (TOLs); (d) dive profile; (e) acceleration along the animal's *x*‐ (surge, forwards‐backwards), *y*‐(sway, side to side), and *z*‐(heave, up and down) axis; (f) jerk calculated as the differential of the three acceleration axes. Periods when the seal is resting at sea, corresponding to low jerk levels, are indicated in the graph. Time is in UTC.

Daily plots were constructed of the TOLs, along with 20 Hz decimated depth, three‐axis acceleration, and RMS jerk (Figure 2). Jerk, J, is an indication of rapid movement of the tag and was computed based on the norm of the differential of the triaxial acceleration following Ydesen et al. (2014), that is:

$${\text{J}}_{\text{t}}={\;\text{f}}_{\text{s}}\surd \left({\left(A_{x,t}-A_{x,t-\text{1}}\right)}^{\text{2}}+{\left(A_{y,t}-A_{y,t-\text{1}}\right)}^{\text{2}}+{\left(A_{z,t}-A_{z,t-\text{1}}\right)}^{\text{2}}\right)$$

where *A_x_*_,_ *_t_* is the triaxial acceleration in m/s^2^ at sample time *t* in the *x*‐axis, and *f*_s_ is the sampling rate. The RMS of *J* was computed over 0.4 s intervals with 50% overlap to produce a time series with 20 Hz sampling rate.

Depth, acceleration, and jerk plots were screened manually for haul out and resting periods at sea. Resting periods at sea were identified as two or more sequential U‐shaped dives, in which the animal showed very little activity in the descending and bottom phases (as assessed from the jerk and accelerometer signal), and a lack of movement at the bottom (i.e., absence of flow noise in the audio data). This behavior was verified as resting on the seafloor by inspection of the camera tag data. Precise start and end times of all resting periods (i.e., the total time from the start of the first dive in a resting bout to the end of the last dive including intervening surface times) were marked in the data. Average submersion time during these resting periods was subsequently determined.

TOL plots (Figure 2) were screened visually for noise events above approx. 70 dB re 1 µPa RMS in one or more third‐octave bands ≥1 kHz. For each event, approx. 10 s of the recording was examined by listening to identify the sound source. If ship noise was encountered, the start and end times of audibility were identified.

For the DTAG‐4, GPS positions were obtained at 2–3 min intervals when the seal was at the surface. After processing, position estimates with an RMS pseudo‐range residual greater than 200 m or with an estimated altitude more than 150 m above or below the WGS‐84 geoid were rejected. The typical pseudo‐range residual of accepted positions was about 20 m. No other track filtering was applied. Automatic Identification System (AIS, i.e., mandatory tracking of all larger ships >300 gross tonnes) records, obtained from The German Federal Waterways and Shipping Administration, were examined to identify all registered vessels within 5 km of each position. The time and range of the closest passage of each of these vessels were recorded. To produce sound exposure plots, the GPS positions were interpolated to 30 s intervals and then plotted as a track colored by the corresponding 1 kHz TOL. This third‐octave band was chosen as being the lowest band that was largely unaffected by low‐frequency flow noise. Given the relatively frequent GPS positions, linear interpolation was used to estimate the positions at 30 s intervals. Outages in GPS measurements lasting more than 10 min were not interpolated.

Videos from the camera tags were viewed in slow motion to classify behaviors into swimming in the water column or along the bottom (possible search behavior), as well as surfacing and resting periods at the bottom. The acceleration data associated with these behaviors were extracted to help interpret similar accelerometer recordings from the DTAGs.

## 3 RESULTS

DTAG deployments yielded 14–21 days of continuous sound and movement data from two gray and three harbor seals, while camera deployments provided 2.5 hr each of combined video and acceleration data on two harbor seals (Table 1).

The audio recordings contained frequent episodes of noise levels with TOLs above 1 kHz exceeding 70 dB re 1 µPa. Figure 2 shows the third‐octave levels for the entire 15.7‐day DTAG‐3 deployment on a gray seal (gs15_139b) in panel a, and in more detail for one day in panel c, which demonstrate frequent fluctuations in noise level. A large proportion of the recorded noise at low frequencies was due to water flow around the tag (flow noise). High broadband sound levels resulted from the seal breaking the surface, bubbles being released from around the tag package or the fur of the seal, from rain, and also close ship passes. Overall, ship noise was audible for 2.2%–20.5% of the time that the four seals, tagged in 2015, spent in water (excl. haul out time, Table 1). This was spread over 17–74 events that lasted 1–330 min, some of which may comprise multiple overlapping vessel passes. Another type of low‐level mechanical noise was also audible over several days in the gs15_139b gray seal recording and may have originated from an offshore wind farm, dredging, or an oil rig.

**Figure 3:**
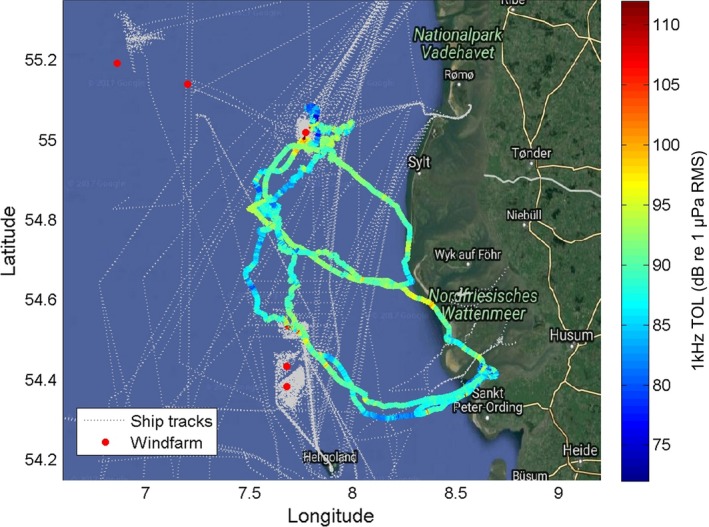
Track of harbor seal hs16_265c, color‐coded with 30‐s averages of third‐octave levels (TOL) in the 1‐kHz‐centered band (see Materials and Methods). Gray dotted lines indicate Automatic Identification System (AIS) tracks of ships that at some point pass within 5 km of the seal (roughly corresponding to the expected range of audibility) and red dots mark the positions of wind farms

In the 2016 harbor seal data (hs16_265c), GPS was recorded successfully along with audio and sensor data providing an opportunity to compare noise exposure events with AIS ship tracks. The average time between GPS positions was a little over two mins throughout the recording time, except for a few longer periods without satellite contact, resulting in 12,972 positions over 21 days. Figure 3 shows the track line color‐coded by sound level. Elevated noise levels (orange/red colors) were in many cases due to ship noise (as confirmed by listening to each high‐noise‐level event). A total of 41 vessel passes were recorded with 1 kHz TOL above 90 dB re 1 µPa RMS (peak 30‐s average), but only in 27 of these cases was an AIS‐registered vessel within 5 km of the animal. This indicates that vessels without AIS were responsible for about one‐third of the high‐level vessel noise exposures experienced by this seal.

**Figure 4:**
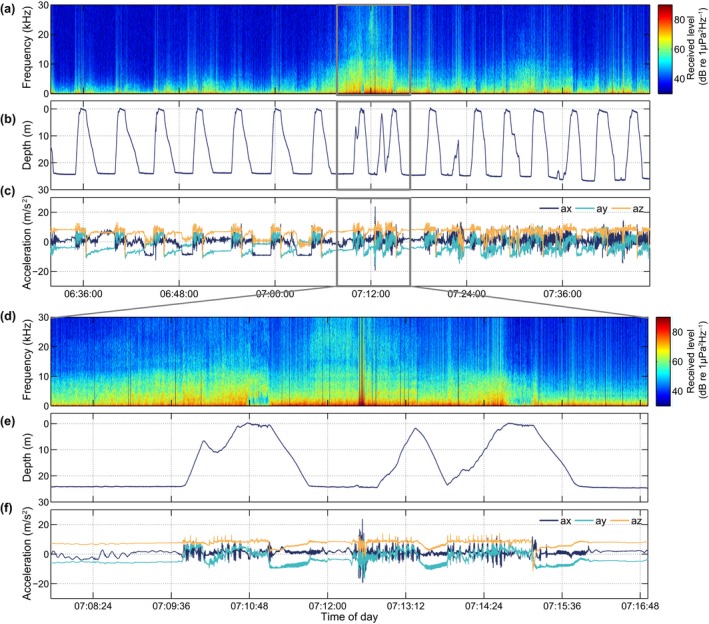
Behavior of gs15_139b before, during, and after the ship encounter on 24 May 2015 indicated in Figure 2f. (a–c) data over a 75‐min period centered on the vessel pass. (a) Received power spectrum density level; (b) dive profile; (c) acceleration profile; (d–f) zoomed‐in view of the same data during 9 min of the vessel pass. Notice the regular dive/resting pattern that is interrupted when ship noise increases

**Figure 5:**
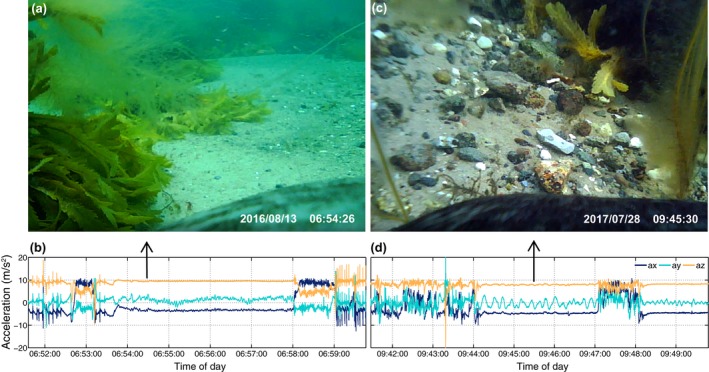
(a, c) Frames from the video recordings obtained with camera tags on two harbor seals (part of the seals are seen in the lower part of each picture); b and d) the associated acceleration profiles, where the arrows indicate the specific time of the snap‐shots. Both images were taken from periods where the seals were resting on the seafloor. The animals are rolling from side to side presumably with the wave cycles (most obvious in d as small oscillations in the y (sway) axis). See the associated video (Supporting information Audio S1) to image (a) in the Supporting Information

Sensor data from the 2015 deployments revealed that the two harbor seals spent 6.6%–42.3% and the two gray seals spent 24%–36% of the recording time hauled out (Table 1). Using audio data, it was possible to differentiate between the seal being completely out of the water or partly submerged: Sounds from water flushing over the tag or the seal lifting its head clear of the water to breathe were associated with partial submergence (Table 1). Resting periods at sea, identified by low acceleration/jerk levels at depth (Figure 2e‐f, Figure 4), were afterward validated by inspection of the camera tag video during intervals with similar low variability of acceleration (Figure 5). Both harbor seals with camera tags exhibited this kind of resting behavior in which the animals lay at the sea floor, rocking slowly with the current (Figure 5 and Video S1). Based on these observations, we found that the harbor seals spent 8.1%–12.4% and the gray seals spent 5.3%–7.2% of their at‐sea time exhibiting this resting behavior. On average, harbor seals were submerged for 4–5.3 min and gray seals for 5.2–6.2 min during resting dives (Table 1).

DTAG recordings from both seal species contained examples of behavioral changes that coincided with vessel encounters. For the gray seal (gs15_139b) depicted in Figure 2b‐c, several ship passages resulted in elevated noise levels (35.5% of the day shown), and at least one of the vessel passes occurred when the seal was in a resting dive, which was subsequently interrupted as shown in Figure 4. In this plot, the animal initially exhibits at‐sea resting behavior. At around 06:44:00, ship noise becomes audible and is clearly visible in the spectral plot by 07:08:00, indicating that the vessel is approaching the animal. Shortly thereafter, at 07:09:50 (Figure 4d–f), the seal breaks off an ascent from an apparently normal resting dive, descents briefly to then resume ascending to the surface. During the next dive (07:12:30), the animal accelerates rapidly at the bottom and makes an interrupted ascent before returning to the surface to breathe. The vessel noise reached a maximum broadband level of 113 dB re 1 µPa RMS (0.1–50 kHz, 1 s average) at 07:12:10 when the seal was still at the bottom. The depth and acceleration data following this vessel encounter (Figure 4b,c) indicate that the seal terminated its resting behavior and began active swimming soon after 07:16:00. Vessel noise ceased to be audible at 09:35:19.

**Figure 6:**
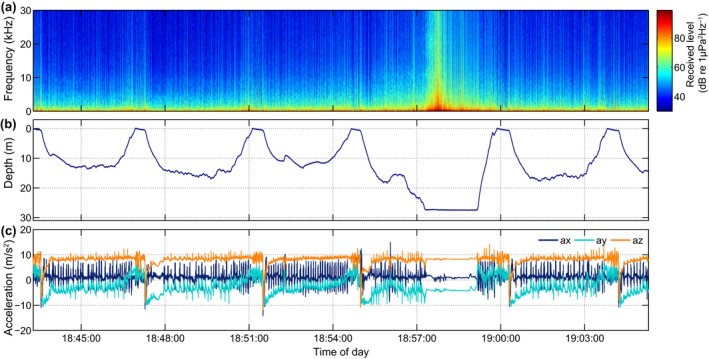
An example of a change in behavior of gray seal gs15_139b presumably caused by vessel disturbance. In the dive beginning at 18:55, the seal descends deeper than in previous dives, presumably to the bottom where the seal remains during the vessel pass. After this fast moving boat passes, the seal resumes its previous diving style. (a) Received power spectrum density level; (b) depth profile; (c) acceleration profile

Another example in which the same animal alters behavior presumably due to vessel disturbance is seen while the seal appears to be performing traveling dives (Figure 6). Here, the animal is diving to approx. 10–15 m depth with continuous flipper stroke‐and‐glide swimming, evident as large oscillations in the x‐ (surge) and y‐axis (sway, Figure 6). During the dive starting at 18:55, the animal suddenly descends to what is presumably the sea bottom (at approx. 27 m depth) where it remains stationary judging by the lack of activity in the accelerometer data. This behavior coincides with a peak in vessel noise from what appears to be a small fast boat given the rapid rise and fall in noise level. In this case, the effect seems to be limited to this particular dive.

**Figure 7:**
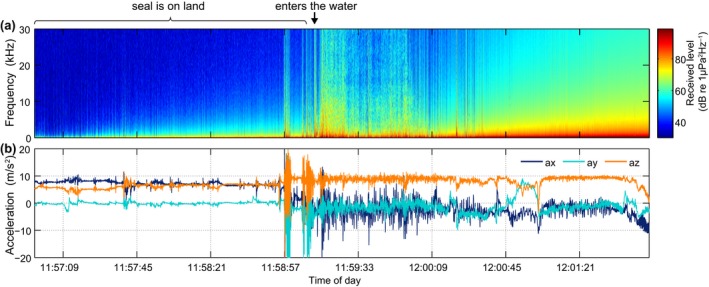
Example of disturbance during haul out of a harbor seal (hs15_069a) on 18 March 2015. (a) Received power spectrum density level; (b) acceleration profile

An example of disturbance during haul out potentially due to the occurrence of a vessel was found in data from harbor seal hs15_069a (Figure 7). Here, the animal is hauled out in the first part of the figure, but at 11:58:57 the animal retreats abruptly into the sea where the audio data reveal high‐level ship noise (this may have been audible to the seal while hauled out, but was not detectable in the tag due to the low sensitivity of the piezo‐ceramic hydrophone in air). Once in the water, the seal swims energetically, as evidenced by large oscillations in the accelerometer signal and high flow noise in the audio, until it surfaces at 12:00:15. The vessel noise disappears when the tag is out of the water but is once again audible when the seal resumes diving (the audio recording can be found in online Supporting Information Audio S1).

## 4 DISCUSSION

Vessel noise is the dominant source of noise pollution in the ocean (Hildebrand, 2009) and may have a particular impact on coastal marine animals whose home ranges overlap strongly with both shipping lanes and recreational boat use. A major challenge in assessing the impact of anthropogenic noise on marine fauna is to quantify both the noise experienced by individual animals and how they respond to it. Here, we demonstrate a method for obtaining these data: High‐resolution multi‐sensor tags (DTAGs) were deployed on harbor and gray seals, providing continuous, broadband audio recordings along with synchronous high‐resolution movement data for up to 21 days. An analysis of these data can be used to establish normal behavioral states and to infer changes in behavior in the context of natural and anthropogenic noise in the environment. The addition of GPS locations in the newest version of the tag provides information on where animals find resources, where they encounter higher levels of noise disturbance, and can aid in identifying specific noise sources such as oil rigs, wind farms, bridges, and ships (i.e., by associating GPS locations with vessel tracks from AIS transmissions).

Using the audio recordings, it was possible to distinguish natural noise sources such as rain from vessel noise and to make a preliminary assessment of the amount of time each noise source was audible. The proportion of time that vessel noise was audible varied widely between animals (2%–20%) likely reflecting differences in the traffic density in the locations visited by animals, with some animals by chance or actively foraging, traveling, or resting in areas with dense ship traffic, for example, commercial fishing grounds. In the example of Figure 4, the vessel pass appeared to provoke an energetic response that terminated a natural resting cycle and precipitated a behavioral change that continued well after the vessel had passed. Similar dive responses to sound stimuli have been seen in elephant seals that changed from ascent to descent and vice‐versa when exposed to noise playbacks made by an acoustic tag attached to their backs (Fregosi et al., 2013). In the example of Figure 7, the animal may have responded to the acoustic or visual cue of the ship while hauled out on land, and again the consequence was a prolonged disruption of resting behavior. Disturbances of seals resting on land have been demonstrated using visual observations and telemetry (Andersen et al., 2012; Edrén et al., 2010; Henry & Hammill, 2001; Jansen, Boveng, Dahle, & Bengtson, 2010; Osinga, Nussbaum, Brakefield, & Udo de Haes, 2012). However, visual contact is inevitably lost soon after seals enter the water making it difficult to assess the duration of response. One of few studies to address this used telemetry to measure the duration and distance covered by seals before returning to a haul out site after being disturbed while hauled‐out (Andersen, Teilmann, Dietz, Schmidt, & Miller, 2014). With the fine‐scale 3D movement data and long duration of the DTAG recordings, it is now possible to study the effects of disturbances in much finer detail along with their frequency of occurrence over several weeks. Thus, the combination of high‐resolution movement and sound recordings provides a powerful link between noise exposures, specific changes in behavior, and their potential energetic costs in terms of swimming effort or time diverted from foraging.

The potential significance of cumulative effects of behavioral responses to disturbance depends upon how often these occur and this has been difficult to assess for any marine mammal species. Although it is attractive to consider AIS ship records as a way to infer vessel noise exposure, close vessel passes deduced from AIS records do not account for all high‐level boat noise events, at least for animals close to the coast where small vessel traffic is more prominent. In the three‐week dataset reported here, only 66% of vessel passes for which the 1 kHz TOL exceeded 90 dB re 1 µPa RMS at the seal were traceable to an AIS reported vessel within 5 km. This highlights the potentially significant, but difficult to predict, contribution of vessel noise from small boats. To account for this, it is important to obtain in situ information about noise exposure for individual animals instead of relying on AIS data, at least for animals close to the coast where small vessel traffic is abundant.

Sound recording tags used on cetaceans have so far been limited by suction cup attachments to about 2 days. Longer duty‐cycled recording durations (up to 8 and 12 days) have been obtained on elephant seals for which a more secure attachment to the fur is possible (e.g., Fregosi et al., 2013; Génin et al., 2015). However, the bandwidth of these recordings was limited to 0.6–16 kHz, the lower end of which overlaps with the frequency range of flow noise making the contribution of vessel noise to the total received level difficult to assess. A narrow bandwidth also limits accurate measurement of noise levels from small boats and larger vessels traveling at high speed which can produce substantial energy at high frequencies (Hermannsen, Beedholm, Tougaard, & Madsen, 2014; Jensen et al., 2009; Wisniewska et al., 2018) that overlap with marine mammal peak hearing sensitivity. As demonstrated here, increasing memory density and new low‐power electronic systems make it possible to achieve both long‐duration and wider recording bandwidth in a miniature biologging tag, deployable on even the smallest pinnipeds. The extended recording time achieved here provides insight into the long‐term behavior of the animals and enables quantification of where, how often, and at what level animals encounter anthropogenic noise sources. The extended recording duration also expands the potential to perform controlled exposure experiments (CEEs) at sea. These experiments have been performed successfully on cetaceans tagged with sound and movement recording tags to assess the impact of specific noise sources (Kvadsheim et al., 2017; Madsen et al., 2006; Miller et al., 2009; Sivle et al., 2012). Still, the short recording duration of these devices meant that little time could be allowed after tag deployment for animals to return to natural behavior and to measure baseline behavior. Longer tag deployments facilitate exclusion of post‐tagging effects, while providing extended baseline data prior to exposure, as well as the possibility of detecting prolonged responses to exposures. The long recording time also opens the potential to perform multiple, widely spaced exposures to the same individuals, which may allow a more thorough evaluation of individual dose‐response functions and potential habituation (Tyack, Gordon, & Thompson, 2004).

The combination of audio, depth, and accelerometer data collected by these long‐duration tags provides a more complete description of behavior than obtained with single‐sensor tags enabling behavioral states and transitions to be detected more precisely. For example, although haul out periods can be detected using just a depth sensor, the accelerometer and audio data allowed us to additionally identify time intervals when the animal was resting on the beach but was partly submerged or flushed during rising tide. Quantifying haul out in this way is less prone to bias compared to using a salt‐water switch in satellite flipper tags, in which the tail must be dry for a minimum time interval to be registered as a haul‐out (Lonergan, Duck, Moss, Morris, & Thompson, 2013). Accurate haul out durations are essential when correcting abundance surveys for the amount of time animals spend on land and thus available for visual detection (Harvey & Goley, 2011; Lonergan et al., 2013).

It has been widely assumed that harbor and gray seals mainly rest at or near the haul out, while floating at the sea surface or during shallow dives (approx. 8 m, Thompson et al., 1991; Russell et al., 2015), these types of resting were also found in the DTAG data. Inspection of the DTAG and video/accelerometry data also revealed frequent resting behavior far offshore at the bottom of U‐shaped dives down to 35 m (Figure 2) as recently suggested from dive profiles by Ramasco et al. (2014). This typically looked like a U‐shaped dive but with low activity during the base of the dive. That these dives comprise resting behavior in harbor seals was confirmed with the video recordings, in which the seal was seen to be lying still or rocking in the current on the bottom. Breath‐hold resting has also been observed in fur seals (*Arctocephalinae*) (Jeanniard‐du‐Dot, Trites, Arnould, Speakman, & Guinet, 2017) and asymmetrical dive profiles (so‐called drift dives) performed by elephant seals are assumed to represent resting behavior (Crocker, Boeuf, & Costa, 1997; Watanabe, Baranov, & Miyazaki, 2015). For harbor and gray seals, U‐shaped dives are typically associated with foraging behavior (Russell et al., 2015; Thompson et al., 1991) and a standard 2D dive profile analysis may have categorized the resting dives found here (e.g., in Figure 4) as U‐shaped foraging dives due to lack of data on fine‐scale movements (Carter et al., 2016). This could lead to overestimation of the number of foraging dives. Although the low activity levels provide clear evidence for resting within dives, these dives also had uniformly asymmetrical profiles with slow descent and fast ascent rates in our data for both species. This suggests the possibility of identifying this behavior from standard 2D dive profiles alone from which they could be excluded from analyses of foraging.

Volpov et al. (2015) deployed head‐mounted 3‐axis accelerometers in free‐ranging Australian fur seals (*Arctocephalus pusillus*) to identify individual attempted prey captures, which was validated using on‐animal video cameras. Prey capture attempts were identified by peaks in the variance of acceleration in either the *x*‐ (surge), *y*‐ (sway), or *z*‐(heave) axis. A similar approach using jerk has been demonstrated with captive harbor seals (Ydesen et al., 2014). However, automatic detection of prey capture attempts from acceleration is complicated by other behaviors that may result in peaks in acceleration (e.g., Figure 4f). From our camera recordings, it appeared that harbor seals mainly searched for prey along the sea bottom, and, as in Volpov et al. (2015), moved their heads from side to side in a swaying motion while swimming energetically close to the bottom. Unfortunately, it was difficult to see whether the seal caught any prey as the camera was mounted on the back of the seal. Hence, future camera/accelerometer deployments with the camera placed further forward will help determine actual prey capture events.

Biologging technology has advanced dramatically over the past decades taking advantage of technological developments in computers, data storage, and battery capacity, and the miniaturization of mobile devices and sensors. These methods are revolutionizing marine mammal science, shedding light on the behaviors of animals and the environments they encounter when out of sight. With this study, we demonstrate the potential use of multi‐week sound and movement recording tags on seals to study exposure to noise, natural undisturbed behavior, and how this behavior may change in response to anthropogenic activities at sea. The combination of long‐duration data with high temporal resolution sensors makes it possible to quantify the frequency and duration of anthropogenic disturbances and how they may affect communication space and essential behaviors like resting and foraging. Conservation of pinnipeds has mainly been focused on their haul out sites, which is due to the limited knowledge of how underwater noise may affect the animals at sea. New biologging techniques allow the quantification of time budgets, and potentially energy budgets, for individual seals, which can then feed into models for population consequences of disturbance (Nabe‐Nielsen et al., 2018). This combination of high‐quality biologging data and modeling is essential for identifying and managing disturbing anthropogenic activities both in time and space, which may compromise the long‐term survival and distribution of marine mammal populations. Management interventions could include reducing impact of vessels by reducing speed, redirecting ship routes, or setting standards for noise emission.

## CONFLICT OF INTEREST

None declared.

## AUTHORS’ CONTRIBUTIONS

LM, MJ, PTM, and JT conceived the ideas. MJ designed the tags which were adapted for recovery by LM and JT. LM, DMW, AvN, US, and JT collected the data; LM, MJ, and DMW analyzed the data. All authors contributed critically to the drafts and gave final approval for publication.

## Data Availability

Datasets generated during the current study is available at Dryad Digital Repository: https://doi.org/10.5061/dryad.8s75sg6.

## References

[ece34923-bib-0001] Aguilar Soto, N. , Johnson, M. , Madsen, P. T. , Tyack, P. L. , Bocconcelli, A. , & Fabrizio Borsani, J. (2006). Does intense ship noise disrupt foraging in deep‐diving Cuvier's beaked whales (*Ziphius cavirostris*)? Marine Mammal Science, 22, 690–699.

[ece34923-bib-0002] Andersen, S. M. , Teilmann, J. , Dietz, R. , Schmidt, N. M. , & Miller, L. A. (2012). Behavioural responses of harbour seals to human‐induced disturbances. Aquatic Conservation: Marine and Freshwater Ecosystems, 22, 113–121. 10.1002/aqc.1244

[ece34923-bib-0003] Andersen, S. M. , Teilmann, J. , Dietz, R. , Schmidt, N. M. , & Miller, L. A. (2014). Disturbance‐induced responses of VHF and satellite tagged harbour seals. Aquatic Conservation: Marine and Freshwater Ecosystems, 24, 712–723. 10.1002/aqc.2393

[ece34923-bib-0004] Arcalís‐Planas, A. , Sveegaard, S. , Karlsson, O. , Harding, K. C. , Wåhlin, A. , Harkonen, T. , & Teilmann, J. (2015). Limited use of sea ice by the Ross seal (*Ommatophoca rossii*), in Amundsen Sea, Antarctica, using telemetry and remote sensing data. Polar Biology, 38, 445–461. 10.1007/s00300-014-1602-y

[ece34923-bib-0005] Blackwell, S. B. , Lawson, J. W. , & Williams, M. T. (2004). Tolerance by ringed seals (*Phoca hispida*) to impact pipe‐driving and construction sounds at an oil production island. The Journal of the Acoustical Society of America, 115, 2346–2357. 15139648 10.1121/1.1701899

[ece34923-bib-0006] Burgess, W. C. , Tyack, P. L. , Le Boeuf, B. J. , & Costa, D. P. (1998). A programmable acoustic recording tag and first results from free‐ranging northern elephant seals. Deep‐Sea Research Part Ii‐Topical Studies in Oceanography, 45, 1327–1351. 10.1016/S0967-0645(98)00032-0

[ece34923-bib-0007] Carter, M. I. D. , Bennett, K. A. , Embling, C. B. , Hosegood, P. J. , & Russell, D. J. F. (2016). Navigating uncertain waters: A critical review of inferring foraging behaviour from location and dive data in pinnipeds. Movement Ecology, 4, 25 10.1186/s40462-016-0090-9 27800161 PMC5080796

[ece34923-bib-0008] Costa, D. P. , Crocker, D. E. , Gedamke, J. , Webb, P. M. , Houser, D. , Blackwell, S. B. , … Le Boeuf, B. J. (2003). The effect of a low‐frequency sound source (Acoustic Thermometry of Ocean Climate) on the diving behavior of juvenile northern elephant seals, *Mirounga angustirostris* . Journal of the Acoustical Society of America, 113, 1155–1165. 12597209 10.1121/1.1538248

[ece34923-bib-0009] Crocker, D. E. , Boeuf, B. J. L. , & Costa, D. P. (1997). Drift diving in female northern elephant seals: Implications for food processing. Canadian Journal of Zoology, 75, 27–39. 10.1139/z97-004

[ece34923-bib-0010] Cunningham, K. A. , & Reichmuth, C. (2016). High‐frequency hearing in seals and sea lions. Hearing Research, 331, 83–91. 10.1016/j.heares.2015.10.002 26519092

[ece34923-bib-0011] Deecke, V. B. , Slater, P. J. B. , & Ford, J. K. B. (2002). Selective habituation shapes acoustic predator recognition in harbour seals. Nature, 420, 171 10.1038/nature01030 12432391

[ece34923-bib-0012] DeRuiter, S. , Southall, B. L. , Calambokidis, J. , Zimmer, W. M. X. , Sadykova, D. , Falcone, E. A. , … Tyack, P. L. (2013). First direct measurements of behavioural responses by Cuvier’s beaked whales to mid‐frequency active sonar. Biological Letters, 9, 20130223 10.1098/rsbl.2013.0223 PMC3730631 23825085

[ece34923-bib-0013] Edrén, S. M. E. , Andersen, S. M. , Teilmann, J. , Carstensen, J. , Harders, P. B. , Dietz, R. , & Miller, L. A. (2010). The effect of a large Danish offshore wind farm on harbor and gray seal haul‐out behavior. Marine Mammal Science, 26, 614–634.

[ece34923-bib-0014] Fletcher, S. , Le Boeuf, B. J. , Costa, D. P. , Tyack, P. L. , & Blackwell, S. B. (1996). Onboard acoustic recording from diving northern elephant seals. The Journal of the Acoustical Society of America, 100, 2531–2539. 10.1121/1.417361 8865656

[ece34923-bib-0015] Fregosi, S. , Klinck, H. , Horning, M. , Mellinger, D. K. , Costa, D. P. , Mann, D. A. , … Huckstadt, L. (2013). Use of an animal‐borne active acoustic tag to conduct minimally‐invasive behavioral response studies. The Journal of the Acoustical Society of America, 134, 4044–4044. 10.1121/1.4830759

[ece34923-bib-0016] Gallon, S. , Bailleul, F. , Charrassin, J. B. , Guinet, C. , Bost, C. A. , Handrich, Y. , & Hindell, M. (2013). Identifying foraging events in deep diving southern elephant seals, Mirounga leonina, using acceleration data loggers. Deep Sea Research Part II: Topical Studies in Oceanography, 88, 14–22. 10.1016/j.dsr2.2012.09.002

[ece34923-bib-0017] Génin, A. , Richard, G. , Jouma'a, J. , Picard, B. , El Ksabi, N. , Vacquié Garcia, J. , & Guinet, C. (2015). Characterization of postdive recovery using sound recordings and its relationship to dive duration, exertion, and foraging effort of southern elephant seals (Mirounga leonina). Marine Mammal Science, 31, 1452–1470.

[ece34923-bib-0018] Götz, T. , & Janik, V. M. (2010). Aversiveness of sounds in phocid seals: Psycho‐physiological factors, learning processes and motivation. Journal of Experimental Biology, 213, 1536–1548. 10.1242/jeb.035535 20400639

[ece34923-bib-0019] Harris, R. E. , Miller, G. W. , & Richardson, W. J. (2001). Seal responses to airgun sounds during summer seismic surveys in the Alaskan Beaufort Sea. Marine Mammal Science, 17, 795–812. 10.1111/j.1748-7692.2001.tb01299.x

[ece34923-bib-0020] Harvey, J. T. , & Goley, D. (2011). Determining a correction factor for aerial surveys of harbor seals in California. Marine Mammal Science, 27, 719–735. 10.1111/j.1748-7692.2010.00446.x

[ece34923-bib-0021] Heaslip, S. G. , Bowen, W. D. , & Iverson, S. J. (2014). Testing predictions of optimal diving theory using animal‐borne video from harbour seals (Phoca vitulina concolor). Canadian Journal of Zoology, 92, 309–318.

[ece34923-bib-0022] Heerah, K. , Hindell, M. , Guinet, C. , & Charrassin, J.‐B. (2014). A new method to quantify within dive foraging behaviour in marine predators. PLoS ONE, 9, e99329 10.1371/journal.pone.0099329 24922323 PMC4055756

[ece34923-bib-0023] Hemilä, S. , Nummela, S. , Berta, A. , & Reuter, T. (2006). High‐frequency hearing in phocid and otariid pinnipeds: An interpretation based on inertial and cochlear constraints. Journal of the Acoustical Society of America, 120, 3463–3466. 10.1121/1.2372712 17225374

[ece34923-bib-0024] Henry, E. , & Hammill, M. O. (2001). Impact of small boats on the haulout activity of harbour seals (*Phoca vitulina*) in Métis Bay, St Lawrence Estuary, Québec, Canada. Aquatic Mammals, 27, 140–148.

[ece34923-bib-0025] Hermannsen, L. , Beedholm, K. , Tougaard, J. , & Madsen, P. T. (2014). High frequency components of ship noise in shallow water: Implications for harbor porpoises (*Phocoena phocoena*). Journal of the Acoustical Society of America, 136, 1640–1653. 25324068 10.1121/1.4893908

[ece34923-bib-0026] Hildebrand, J. (2005). Impacts of anthropogenic sound In Reynolds J. E., Perrin W. F., Reeves R. R., Montgomery S., & Ragen T. J. (Eds.), Marine mammal research: Conservation beyond Crisis (pp. 101–124). Baltimore, MD: The Johns Hopkins University Press.

[ece34923-bib-0027] Hildebrand, J. A. (2009). Anthropogenic and natural sources of ambient noise in the ocean. Marine Ecology Progress Series, 395, 5–26. 10.3354/meps08353

[ece34923-bib-0028] Jansen, J. K. , Boveng, P. L. , Dahle, S. P. , & Bengtson, J. L. (2010). Reaction of Harbor Seals to Cruise Ships. Journal of Wildlife Management, 74, 1186–1194. 10.1111/j.1937-2817.2010.tb01239.x

[ece34923-bib-0029] Jeanniard‐du‐Dot, T. , Trites, A. W. , Arnould, J. P. Y. , Speakman, J. R. , & Guinet, C. (2017). Activity‐specific metabolic rates for diving, transiting, and resting at sea can be estimated from time–activity budgets in free‐ranging marine mammals. Ecology and Evolution, 7, 2969–2976. 10.1002/ece3.2546 28479996 PMC5415512

[ece34923-bib-0030] Jeffries, S. J. , Brown, R. F. , & Harvey, J. T. (1993). Techniques for capturing, handling and marking harbour seals. Aquatic Mammals, 19, 21–25.

[ece34923-bib-0031] Jensen, F. H. , Bejder, L. , Wahlberg, M. , Aguilar Soto, N. , Johnson, M. , & Madsen, P. T. (2009). Vessel noise effects on delphinid communication. Marine Ecology Progress Series, 395, 161–175. 10.3354/meps08204

[ece34923-bib-0032] Johnson, M. , Aguilar Soto, N. , & Madsen, P. T. (2009). Studying the behaviour and sensory ecology of marine mammals using acoustic recording tags: A review. Marine Ecology Progress Series, 395, 55–73. 10.3354/meps08255

[ece34923-bib-0033] Johnson, M. , Partan, J. , & Hurst, T. (2013). Low complexity lossless compression of underwater sound recordings. The Journal of the Acoustical Society of America, 133, 1387–1398. 10.1121/1.4776206 23464010

[ece34923-bib-0034] Johnson, M. , & Tyack, P. L. (2003). A digital acoustic recording tag for measuring the response of wild marine mammals to sound. IEEE Journal of Oceanic Engineering, 28, 3–12. 10.1109/JOE.2002.808212

[ece34923-bib-0035] Kastelein, R. , Helder‐Hoek, L. , Gransier, R. , Terhune, J. M. , Jennings, N. , & De Jong, C. A. F. (2015). Hearing thresholds of harbor seals (*Phoca vitulina*) for playbacks of seal scarer signals, and effects of the signals on behaviour. Hydrobiologia, 756, 75–88.

[ece34923-bib-0036] Kastelein, R. A. , van der Heul, S. , Terhune, J. M. , Verboom, W. C. , & Triesscheijn, R. J. V. (2006a). Deterring effects of 8–45 kHz tone pulses on harbour seals (*Phoca vitulina*) in a large pool. Marine Environmental Research, 62, 356–373. 16870247 10.1016/j.marenvres.2006.05.004

[ece34923-bib-0037] Kastelein, R. A. , van der Heul, S. , Verboom, W. C. , Triesscheijn, R. J. V. , & Jennings, N. V. (2006b). The influence of underwater data transmission sounds on the displacement behaviour of captive harbour seals (*Phoca vitulina*). Marine Environmental Research, 61, 19–39. 16038972 10.1016/j.marenvres.2005.04.001

[ece34923-bib-0038] Kvadsheim, P. H. , DeRuiter, S. , Sivle, L. D. , Goldbogen, J. , Roland‐Hansen, R. , Miller, P. J. O. , … Southall, B. (2017). Avoidance responses of minke whales to 1–4 kHz naval sonar. Marine Pollution Bulletin, 121, 60–68. 10.1016/j.marpolbul.2017.05.037 28552251

[ece34923-bib-0039] Lonergan, M. , Duck, C. , Moss, S. , Morris, C. , & Thompson, D. (2013). Rescaling of aerial survey data with information from small numbers of telemetry tags to estimate the size of a declining harbour seal population. Aquatic Conservation: Marine and Freshwater Ecosystems, 23, 135–144. 10.1002/aqc.2277

[ece34923-bib-0040] Madsen, P. T. , Johnson, M. , Miller, P. J. O. , Aguilar Soto, N. , Lynch, J. , & Tyack, P. (2006). Quantitative measures of air‐gun pulses recorded on sperm whales (*Physeter* *macrocephalus*) using acoustic tags during controlled exposure experiments. The Journal of the Acoustical Society of America, 120, 2366–2379. 17069331 10.1121/1.2229287

[ece34923-bib-0041] Mathevon, N. , Casey, C. , Reichmuth, C. , & Charrier, I. (2017). Northern Elephant Seals Memorize the Rhythm and Timbre of Their Rivals' Voices. Current Biology, 27, 2352–2356.e2352. 10.1016/j.cub.2017.06.035 28736171

[ece34923-bib-0042] McCauley, D. J. , Pinsky, M. L. , Palumbi, S. R. , Estes, J. A. , Joyce, F. H. , & Warner, R. R. (2015). Marine defaunation: Animal loss in the global ocean. Science, 347, 1255641 10.1126/science.1255641 25593191

[ece34923-bib-0043] McConnell, B. , Beaton, R. , Bryant, E. , Hunter, C. , Lovell, P. , & Hall, A. (2004). Phoning home‐a new GSM mobile phone telemetry system to collect mark‐recapture data. Marine Mammal Science, 20, 274–283. 10.1111/j.1748-7692.2004.tb01156.x

[ece34923-bib-0044] Miller, P. J. O. , Johnson, M. P. , Madsen, P. T. , Biassoni, N. , Quero, M. , & Tyack, P. L. (2009). Using at‐sea experiments to study the effects of airguns on the foraging behavior of sperm whales in the Gulf of Mexico. Deep Sea Research Part I: Oceanographic Research Papers, 56, 1168–1181. 10.1016/j.dsr.2009.02.008

[ece34923-bib-0045] Nabe‐Nielsen, J. , Beest, F. M. , Grimm, V. , Sibly, R. M. , Teilmann, J. , & Thompson, P. M. (2018). Predicting the impacts of anthropogenic disturbances on marine populations. Conservation Letters, 11(5), e12563 10.1111/conl.12563

[ece34923-bib-0046] Nowacek, D. P. , Johnson, M. P. , & Tyack, P. L. (2004). North Atlantic right whales (*Eubalaena glacialis*) ignore ships but respond to alerting stimuli. Proceedings of the Royal Society of London. Series B: Biological Sciences, 271, 227–231. 15058431 10.1098/rspb.2003.2570 PMC1691586

[ece34923-bib-0047] Nowacek, D. P. , Thorne, L. H. , Johnston, D. W. , & Tyack, P. L. (2007). Responses of cetaceans to anthropogenic noise. Mammal Review, 37, 81–115. 10.1111/j.1365-2907.2007.00104.x

[ece34923-bib-0048] Osinga, N. , Nussbaum, S. B. , Brakefield, P. M. , & Udo de Haes, H. A. (2012). Response of common seals (Phoca vitulina) to human disturbances in the Dollard estuary of the Wadden Sea. Mammalian Biology – Zeitschrift Für Säugetierkunde, 77, 281–287. 10.1016/j.mambio.2012.02.005

[ece34923-bib-0049] Ramasco, V. , Biuw, M. , & Nilssen, K. T. (2014). Improving time budget estimates through the behavioural interpretation of dive bouts in harbour seals. Animal Behaviour, 94, 117–134. 10.1016/j.anbehav.2014.05.015

[ece34923-bib-0050] Richardson, W. J. , Greene, C. R. , Malme, C. I. , & Thomson, D. H. (1995). Marine mammals and noise. San Diego: Academic Press.

[ece34923-bib-0051] Russell, D. J. F. , Hastie, G. D. , Thompson, D. , Janik, V. M. , Hammond, P. S. , Scott‐Hayward, L. A. S. , … McConnell, B. J. (2016). Avoidance of wind farms by harbour seals is limited to pile driving activities. Journal of Applied Ecology, 56, 1642–1652. 10.1111/1365-2664.12678 PMC5111737 27867217

[ece34923-bib-0052] Russell, D. J. F. , McClintock, B. T. , Matthiopoulos, J. , Thompson, P. M. , Thompson, D. , Hammond, P. S. , … McConnell, B. J. (2015). Intrinsic and extrinsic drivers of activity budgets in sympatric grey and harbour seals. Oikos, 124, 1462–1472. 10.1111/oik.01810

[ece34923-bib-0053] Schusterman, R. J. , Levenson, D. H. , Reichmuth, C. J. , & Southall, B. L. (2000). Why pinnipeds don't echolocate. Journal of the Acoustical Society of America, 107, 2256–2264. 10.1121/1.428506 10790051

[ece34923-bib-0054] Sivle, L. D. , Kvadsheim, P. H. , Fahlman, A. , Lam, F. P. A. , Tyack, P. L. , & Miller, P. J. O. (2012) Changes in dive behavior during naval sonar exposure in killer whales, long‐finned pilot whales, and sperm whales. Frontiers in Physiology, 3, 400. 23087648 10.3389/fphys.2012.00400 PMC3468818

[ece34923-bib-0055] Thompson, D. , Hammond, P. S. , Niceolas, K. S. , & Fedak, M. A. (1991). Movements, diving and foraging behaviour of grey seals (*Halichoerus grypus*). Journal of Zoology, 224, 223–232.

[ece34923-bib-0056] Tyack, P. L. , Gordon, J. , & Thompson, D. (2004). Controlled exposure experiments to determine the effects of noise on marine mammals. Marine Technology Society Journal, 37, 41–53. 10.4031/002533203787537087

[ece34923-bib-0057] Tyack, P. L. , Zimmer, W. M. X. , Moretti, D. , Southall, B. L. , Claridge, D. E. , Durban, J. W. , … Boyd, I. L. (2011). Beaked whales respond to simulated and actual navy sonar. PLoS ONE, 6, e17009 10.1371/journal.pone.0017009 21423729 PMC3056662

[ece34923-bib-0058] van Beest, F. M. , Teilmann, J. , Hermannsen, L. , Galatius, A. , Mikkelsen, L. , Sveegaard, S. , … Nabe‐Nielsen, J. (2018). Fine‐scale movement responses of free‐ranging harbour porpoises to capture, tagging and short‐term noise pulses from a single airgun. Royal Society Open Science, 5, 170110 10.1098/rsos.170110 29410789 PMC5792866

[ece34923-bib-0059] Van Parijs, S. M. , Hastie, G. D. , & Thompson, P. M. (1999). Geographical variation in temporal and spatial vocalization patterns of male harbour seals in the mating season. Animal Behaviour, 58, 1231–1239. 10.1006/anbe.1999.1258 10600144

[ece34923-bib-0060] Volpov, B. L. , Hoskins, A. J. , Battaile, B. C. , Viviant, M. , Wheatley, K. E. , Marshall, G. , … Arnould, J. P. Y. (2015). Identification of Prey Captures in Australian Fur Seals (*Arctocephalus pusillus* doriferus) Using Head‐Mounted Accelerometers: Field Validation with Animal‐Borne Video Cameras. PLoS ONE, 10, e0128789 10.1371/journal.pone.0128789 26107647 PMC4479472

[ece34923-bib-0061] Watanabe, Y. Y. , Baranov, E. A. , & Miyazaki, N. (2015). Drift dives and prolonged surfacing periods in Baikal seals: Resting strategies in open waters? The Journal of Experimental Biology, 218, 2793. 26139663 10.1242/jeb.125898

[ece34923-bib-0062] Wisniewska, D. M. , Johnson, M. , Teilmann, J. , Siebert, U. , Galatius, A. , Dietz, R. , & Madsen, P. T. (2018). High rates of vessel noise disrupt foraging in wild harbour porpoises (*Phocoena phocoena*). Proceedings of the Royal Society B: Biological Sciences, 285. 10.1098/rspb.2017.2314 PMC5829196 29445018

[ece34923-bib-0063] Wisniewska, D. M. , Johnson, M. , Teilmann, J. , Rojano‐Donate, L. , Shearer, J. , Sveegaard, S. , … Madsen, P. T. (2016). Ultra‐high foraging rates of harbor porpoises make them vulnerable to anthropogenic disturbance. Current Biology, 26, 1441–1446. 10.1016/j.cub.2016.03.069 27238281

[ece34923-bib-0064] Ydesen, K. , Wisniewska, D. M. , Hansen, J. D. , Beedholm, K. , Johnson, M. , & Madsen, P. T. (2014). What a jerk: Prey engulfment revealed by high‐rate, super‐cranial accelerometry on a harbour seal (Phoca vitulina). Journal of Experimental Biology, 217, 2239–2243. 10.1242/jeb.100016 24737765

